# Glioblastoma hijacks microglial gene expression to support tumor growth

**DOI:** 10.1186/s12974-020-01797-2

**Published:** 2020-04-16

**Authors:** Sybren L. N. Maas, Erik R. Abels, Lieke L. Van De Haar, Xuan Zhang, Liza Morsett, Srinjoy Sil, Joana Guedes, Pritha Sen, Shilpa Prabhakar, Suzanne E. Hickman, Charles P. Lai, David T. Ting, Xandra O. Breakefield, Marike L. D. Broekman, Joseph El Khoury

**Affiliations:** 1 Departments of Neurology and Radiology, Massachusetts General Hospital, Harvard Medical School, Boston, MA 02129 USA; 2 Department of Neurosurgery, UMC Utrecht Brain Center, University Medical Center, Utrecht University, 3584 CX Utrecht, The Netherlands; 3 Cancer Center, Massachusetts General Hospital, Harvard Medical School, Boston, MA 02129 USA; 4 Center for Immunology & Inflammatory Diseases, Massachusetts General Hospital, Harvard Medical School, Boston, MA 02129 USA; 5 Center for Neuroscience and Cell Biology, University of Coimbra, 3004-517 Coimbra, Portugal; 6 Department of Medicine, Massachusetts General Hospital, Harvard Medical School, Boston, MA 02129 USA; 7 Institute of Atomic and Molecular Sciences/Academia Sinica, 10617 Taipei, Taiwan; 8 Department of Neurosurgery, Leiden University Medical Center, 2300 RC Leiden, The Netherlands; 9 Department of Neurosurgery, Haaglanden Medical Center, 2512 VA The Hague, The Netherlands

**Keywords:** Glioblastoma, Glioma, Microglia, Extracellular vesicles, Exosomes, Microvesicles, Macrophages, Sensome, RNASeq, TGF-β

## Abstract

**Background:**

Glioblastomas are the most common and lethal primary brain tumors. Microglia, the resident immune cells of the brain, survey their environment and respond to pathogens, toxins, and tumors. Glioblastoma cells communicate with microglia, in part by releasing extracellular vesicles (EVs). Despite the presence of large numbers of microglia in glioblastoma, the tumors continue to grow, and these neuroimmune cells appear incapable of keeping the tumor in check. To understand this process, we analyzed gene expression in microglia interacting with glioblastoma cells*.*

**Methods:**

We used RNASeq of isolated microglia to analyze the expression patterns of genes involved in key microglial functions in mice with glioblastoma. We focused on microglia that had taken up tumor-derived EVs and therefore were within and immediately adjacent to the tumor.

**Results:**

We show that these microglia have downregulated expression of genes involved in sensing tumor cells and tumor-derived danger signals, as well as genes used for tumor killing. In contrast, expression of genes involved in facilitating tumor spread was upregulated. These changes appear to be in part EV-mediated, since intracranial injection of EVs in normal mice led to similar transcriptional changes in microglia. We observed a similar microglial transcriptomic signature when we analyzed datasets from human patients with glioblastoma.

**Conclusion:**

Our data define a microglia_Glioblastoma_ specific phenotype, whereby glioblastomas have hijacked gene expression in the neuroimmune system to favor avoiding tumor sensing, suppressing the immune response, clearing a path for invasion, and enhancing tumor propagation. For further exploration, we developed an interactive online tool at http://www.glioma-microglia.com with all expression data and additional functional and pathway information for each gene.

## Background

Harnessing the power of the immune system to treat cancer has gained significant momentum in recent years. Glioblastomas are diffusely infiltrating tumors of the brain. Because of their invasive nature, total neurosurgical resection of glioblastomas is not possible, resulting in tumor recurrence even following chemo- and radiotherapy [1]. Therefore, new effective treatment strategies for glioblastomas are desperately needed, including therapies utilizing the patients’ own immune system [2]. Understanding how glioblastoma cells interact with the immune system is the key to developing immune-based treatments for this tumor [2].

Glioblastomas recruit neighboring resident microglia through the secretion of various chemokines and cytokines [3, 4]. These microglia together with infiltrating monocytes and macrophages can make up to 44% of the glioblastoma mass [5, 6]. However, in spite of the presence of large numbers of microglia, monocytes, and macrophages in glioblastoma, the tumors continue to grow, and immune cells appear incapable of controlling such growth. It is accepted that glioblastoma-associated microglia, monocytes, and macrophages play a role in promoting tumor growth [7, 8]. Indeed, depletion of these cells results in reduced glioblastoma invasion and growth in organotypic brain slices and in vivo [9, 10]. While the evidence that supports this assertion is growing, the exact pathways involved in this tumor-supportive process have not been characterized. Furthermore, the effect(s) of microglia, monocytes, and macrophages that are within the tumor environs versus those in other areas of the tumor-bearing brain but distant from the tumor have not been investigated.

Tumor cells can alter their milieu in part by releasing extracellular vesicles (EVs), including exosomes and microvesicles [11–13]. EVs are a heterogeneous collection of membrane-bound carriers with complex cargoes, including proteins, lipids, and nucleic acids [13–16]. Tumor-derived EV uptake by microglia leads to changes in expression of some genes in these cells as established in vitro [17, 18]. We have previously visualized such interactions both in vitro and in vivo using a syngeneic mouse glioblastoma model expressing palmitoylated green or red fluorescent proteins (palmGFP and palmtdTomato, respectively) [18–20]. These palmitoylated fluorescent proteins label membranes of tumor cells as well as EVs produced by them (e.g., EV-GFP) [20]. This model allowed us to visualize and isolate microglia, monocytes, and macrophages that had taken up tumor-derived EVs in vivo and are therefore closely interacting with glioblastoma cells. In the work presented here, we isolated these microglia, monocytes, and macrophages by fluorescence activated cell sorting (FACS) and analyzed their transcriptomes using bulk RNAseq. To facilitate future analysis of these transcriptomes, we developed an interactive online tool with additional functional and pathway information linked to each gene. To illustrate the usefulness of our dataset and online tool, we performed a focused analysis of microglia. We found that EV-GFP^pos^ microglia (i.e., present within the tumor) have dysregulated expression of genes in the homeostatic TGF-β pathway suggesting a disease specific non-homeostatic phenotype in glioma microglia. Furthermore, genes involved in sensing tumor cells, host defense, and those involved in tumor killing were downregulated, whereas those involved in facilitating tumor spread were upregulated. The evoked role of tumor-derived EVs in this microglial transformation was supported by finding similar changes in microglia isolated after uptake of glioma-derived EVs injected intracranially into the brain. Our results were further validated when we analyzed existing bulk and single-cell sequencing datasets of human glioblastoma-associated microglia and found that these microglia displayed similar alterations as observed in the mice. Taken together, these data identify specific changes in the transcriptome of microglia in the presence of glioblastoma that support tumor growth.

## Materials and methods

### Mice

Animal experimentation was approved by the Massachusetts General Hospital Institution Animal Care and Use Committee. C57BL/6 mice (Charles River Laboratories) were crossed with homozygous C57/BL6.CCR2^RFP/RFP^ knock-in mice [21] to generate heterozygous C57BL6.CCR2^RFP/WT^ knock-in mice. Mice were maintained under a 12-h light/dark cycle with access to water and food. Adult mice ranging from 12–18 weeks were used in this study. Male and female mice were randomly assigned to experimental groups. Mice had similar tumor sizes. RNAseq of microglia from male and female animals showed no differences in expression between males and females (data not shown). The 4-week time point was chosen as this is the time point at which mice implanted with GL261 cells first start to develop physical signs and have to be sacrificed per animal welfare guidelines.

### Cell culture

Mouse glioblastoma cell-line GL261 wildtype (NCI Tumor Repository) was cultured in Dulbecco’s modified Eagle’s medium (DMEM) (Corning) supplemented with 10% fetal bovine serum (FBS) (Gemini Bioproducts), penicillin (100 units.ml^−1^), and streptomycin (100 μg.ml^−1^) (Corning). Cells were cultured at 37 °C in a 5% CO2 humidified incubator. Cells were periodically tested for mycoplasma contamination and found negative.

### Stable transduction reporter

To introduce reporter molecules, the mouse glioblastoma cell-line GL261 wildtype (NCI Tumor Repository) was stably transduced using a CSCW2 lentiviral vector [22] encoding a Gaussia luciferase transmembrane biotin acceptor domain fusion protein (GlucB) and GFP separated by an internal ribosome entry site (IRES) domain [23]. A second transduction was performed using a CSCW2 lentiviral vector encoding palmitoylated GFP for pan membrane-associated GFP expression, including in membrane particles released by these tumor cells [20, 24]. Selection and validation of viral transduction and reporter expression, resulting in the generation of GL261.GlucB-IRES-GFP.palmGFP (GL261.BpalmGFP) cells, were done based on GFP expression using FACS (BD FACSAria II SORP Cell Sorter).

### Intracranial tumor implantation

After anesthetizing the animals using 70 μl of a mixture of ketamine (Bioniche Pharma) (17.5 mg.ml^−1^) and xylazine (Santa Cruz Biotechnology) (2.5 mg.ml^−1^), C57BL6.CCR2^RFP/wt^ adult mice (12–18 weeks old) were implanted in the striatum with 1 × 10^5^ GL261.BpalmGFP or GL261 wildtype cells in 2 μl plain DMEM using a stereotactic frame. Cells were implanted using the coordinates from lambda—2 mm anterior, 0.5 mm left, and a depth of 2.5 mm from the skull. Four weeks after implantation, the mice were deeply anesthetized with 120 μl of a mixture of ketamine (17.5 mg.ml^−1^) and xylazine (2.5 mg.ml^−1^) followed by transcardial perfusion with 50 ml PBS for FACS or 4% PFA (VWR) for immunohistochemistry using a perfusion pump (Minipump Variable Flow, Fisher Scientific).

### EV isolation and intracranial injection

EVs were isolated from supernatant of GL261.BpalmGFP cultured for 48 h in DMEM with penicillin (100 units.ml^−1^) and streptomycin (100 μg.ml^−1^) (Corning) and EV-depleted FBS. FBS was depleted of EVs by overnight (16 h) ultracentrifugation at 200.000×*g* (*k*-factor 110.5). EV isolation was done using differential ultracentrifugation protocol consistent of centrifugation of supernatant at 300×*g* for 10 min, 2000×*g* 10 min, filtering through 0.8 μm filter (Sigma), and 100.000×*g* (*k*-factor of 220.1) 120 min in Quick-Seal® Polypropylene Tubes (Beckman) using Type 70 Ti in Optima^TM^ XE ultracentrifuge (Beckman) to pellet EVs. EV pellets were concentrated by centrifugation at 100.000×*g* (*k*-factor of 190.7) for 120 min in Thinwall Polypropylene Tubes (Beckman) using MLS-50 Swinging-Bucket Rotor (Beckman) in an Optima^TM^ MAX-XP Ultracentrifuge (Beckman). Pelleted EVs were resuspended in PBS, and subsequent characterization of EV pellet was performed by size distribution analysis using nanoparticle tracking analysis (Malvern) and western blot analysis. For western bolt analysis, EV pellets and cells were resuspended in RIPA buffer. Equal amount of protein as measured by Pierce BCA protein assay (Thermo Fisher) was loaded and ran on 10% SDS-PAGE gel (Thermo Fisher). Proteins were transferred onto nitrocellulose membrane and probed for ALIX (Santa Cruz, sc-53538, 1:200), TSG101 (Abcam, ab125011, 1:500), Flotillin-1 (Abcam, ab133497, 1:500), GAPDH (Millipore, CB1001, 1:1000), and GFP (Thermo Fisher, A-11120, 1:1000).

EV or carrier fluid (PBS) was injected intracranial following identical procedures as described in intracranial tumor implantation method section. Using NTA 2.2 with shutter set at 1000 and gain at 400, a 1 to 500 dilution of EV concentrate was measured with > 1000 completed tracks [25]. A total of 3 μl with a concentration of 1.4e12 particles.ml^−1^ was injected. Microglia were isolated 16 h after injection of EV or DPBS following procedures as described in methods sections harvesting of brains and preparation of single-cell suspensions and cell staining and FACS.

### Immunohistochemistry

Brains were collected and placed in 4% PFA for 24 h and subsequently placed in 25% sucrose for 48 h. The brains were then frozen in optimal cutting temperature compound (OCT) media (Sakura) in a dry ice bath containing 2-methyl-butanol. Twelve micro cryosections were prepared, placed on glass slides, and stored at − 80 °C. For processing, sections were washed for 10 min in PBS and permeabilized with 0.5% Triton-X PBS for 1 h at room temperature. Sections were blocked for 1 h at room temperature using 5% Normal Goat Serum (NGS) (Abcam) in PBS. Subsequently, the sections were labeled with a primary goat antibody and blocked using 5% Bovine Serum Albumin (BSA; Sigma-Aldrich) in PBS. Primary antibodies were diluted in 1.5% NGS or 1.5% BSA. Slides were then incubated with primary antibody solution overnight at 4 °C. After incubation, slides were washed 3 × 10 min in PBS. The secondary antibodies were diluted in 1.5% NGS or 1.5% BSA. Sections were then incubated with secondary antibody solution for 1 h at room temperature and subsequently washed 3 × 10 min using PBS. DAPI (0.1 μg.ml^−1^, Thermo Fisher) staining was performed for 30 min at room temperature. Next, the slides were washed for 10 min using PBS. Sections were mounted using ProLong Diamond Antifade Mountant (Thermo Fisher). Primary antibodies used were goat-anti-mouse ARG1 (Santa Cruz Biotechnology, sc18354, 1:200), goat-anti-mouse CD74 (Santa Cruz Biotechnology, sc5438, 1:200), rabbit-anti-mouse IBA1 (Wako, 019-19741, 1:1000), and mouse-anti-GFP tag antibody (Thermo Fisher, A-11120, 1:200). Secondary antibodies were donkey-anti-goat IgG Alexa Fluor 647 (Thermo Fisher, A21447, 1:500), donkey-anti-rabbit IgG Alexa Fluor 405 (Thermo Fisher, A31556, 1:500), and goat-anti-mouse IgG Alexa Fluor 488 (Thermo Fisher, A31560, 1:500).

### Microscopy

Fluorescence microscopy images were acquired on the Zeiss Axio Imager M2 (Carl Zeiss). Confocal images were obtained using the Zeiss LSM 710 inverted confocal microscope.

### Harvesting of brains and preparation of single-cell suspensions

After anesthetizing and perfusing with PBS, brains were removed and processed into single-cell suspension as described [26]. Briefly, brains were cut into small pieces and placed into a GentleMacs™ C-tube (Miltenyi Biotech, San Diego, CA, USA) with Roswell Park Memorial Institute (RPMI) 1640 with l-glutamine (no phenol red) medium (Fisher Scientific) containing dispase (2 U.ml^−1^) (Corning) and collagenase type 3 at a final concentration of 200 U.ml^−1^ (Worthington Biochemicals). The resulting mixtures were processed using the gentleMACS Dissociator (Miltenyi Biotech) on the brain program settings according to manufacturer’s directions. Thus, the brains were subjected to three rounds of dissociation each followed by a period of incubation at 37 °C for 10 min. DNase I grade II (Roche Applied Science) was added to a final concentration of 40 U.ml^−1^ and incubated for an additional 10 min before the final round of dissociation. After dissociation steps, PBS/EDTA containing 5% FBS was added to inactivate the enzyme mixture, and brain pieces were gently triturated gently, passed through a 100-μm filter (Fisher Scientific) and centrifuged at 400×*g* for 10 min. Cell pellets were resuspended in 10.5 ml RPMI/l-glutamine, mixed gently with 4.5 ml physiologic Percoll® (Sigma Aldrich), and centrifuged at 850×*g* without brake for 40 min. The subsequent pellets were then rinsed in PBS and centrifuged again at 400×*g* for 10 min. Red blood cells in the pellets were lysed using RBC lysis buffer (Boston BioProducts) for 2 min at room temperature followed by a washing step using RPMI/l-glutamine medium. The final cell suspensions were then resuspended in PBS with 0.2% FBS or in DPBS, 1× without calcium (Ca^2+^) and magnesium (Mg^2+^) (Corning) supplemented with 2 mM EDTA (Thermo Fisher), and 0.5% BSA (Sigma Aldrich), followed by staining and FACS. The interval between perfusion to FACS was approximately 5 h.

### Cell staining and FACS

To block non-specific binding of immunoglobulin to the Fc receptors, cells in suspension were incubated for 10 min on ice with TruStain fcX™ (anti-mouse CD16/32, BioLegend, #101319, clone 93, 1:100). Cell identification was based on levels of expression of CD45 and CD11b (microglia), CD45, CD11b, F4/80, Ly6C, and CCR2 (monocytes/macrophages). For microglia, we used anti-CD45-pacificBlue (BioLegend, #103125, clone 30-F11, 1:100) and anti-CD11b-Alexa647 (BioLegend, #101220, clone M1/70, 1:100) for tumor bearing mice. For monocytes/macrophages, anti-CD45-pacificBlue (BioLegend, #103125, clone 30-F11, 1:100), anti-CD11b-PE-Cy7 (BioLegend, #101215, clone M1/70, 1:100), anti-Ly6C-BV605 (BioLegend, #128035, clone HK1.4, 1:500), and anti-F4/80-APC (BioLegend, #123115, clone BM8, 1:75) were used. Cells were stained for 30 min on ice with gentle mixing every 10 min by pipetting the mixture up and down. To remove unbound antibodies, cells were centrifuged at 400×*g* for 8 min, resuspended in 0.2% FBS in PBS, and passed through a 35-μm nylon mesh strainer (BD Falcon). Cells were than sorted using a BD FACSAria II SORP Cell Sorter.

### RNA isolation and preparation for RNA sequencing

Cells isolated from brains in all experiments were directly sorted into 1.5 ml Eppendorf (Hauppauge) tubes containing 350 μl RLT Plus lysis buffer (Qiagen) at 4 °C. After FACS was completed, the tubes were weighed, and additional RLT Plus was added to the 1.5 ml Eppendorf if the sorted volume was larger than 50 μl at a ratio of a maximum of 50 μl 0.2% FBS PBS to 350 μl RLT Plus buffer. 2-Mercaptoethanol (Sigma) was added to the tubes at a ratio of 10 μl per 1 ml of RLT buffer, and RNA was then isolated using the RNeasy Plus Micro kit (Qiagen) and using the total RNA isolation protocol. Eluted RNA was aliquoted and stored at − 80 °C. Before preparation of cDNA fragments for RNA sequencing, RNA concentrations and quality were determined using the Agilent 2100 Bioanalyzer (Agilent Technologies) Pico-chips. cDNA for RNA sequencing was synthesized from RNA aliquots using the SMARTer Ultra Low Input RNA Kit for Sequencing–v3 (Clontech Takara) according to the manufacturer’s protocol. A total of 500 pg RNA were used for subsequent library generation. One microliter of a 1:50,000 dilution of ERCC RNA Spike-In Mix (Life Technologies) was added to each sample. Then, first-strand synthesis and tailing of RNA molecules were performed using 3′-SMART CDS primer II A (selecting for poly-A-tails) followed by extension and template switching by reverse transcriptase. Amplified cDNA was purified with 1x Agencourt AMPure XP beads (Beckman Coulter), in accordance with the SMARTer protocol. The eluted cDNA was stored at − 20 °C. The Nextera® XT DNA Library Preparation kit (Illumina) was used for sample barcoding and fragmentation according to the manufacturer’s protocol. cDNA samples were thawed, and a total of 1 ng of amplified cDNA was used for the enzymatic tagmentation followed by 12 cycles of amplification and unique dual-index barcoding of individual libraries. PCR product was purified with 1.8x Agencourt AMPure XP beads as detailed in the Nextera XT protocol, omitting the bead-based library normalization step. Library validation and quantification was performed by quantitative PCR using the SYBR® FAST Universal qPCR Kit (KAPA Biosystems). The individual libraries were pooled with equal concentrations, and the pool concentration was re-determined using the KAPA SYBR® FAST Universal qPCR Kit. The pool of libraries was subsequently diluted, denatured, and loaded onto the NextSeq 500 sequencer (Illumina) according to the manufacturer’s guidelines with the addition of 1% PhiX Sequencing Control V3 (Illumina). A NextSeq 500/550 High Output v2 kit (150 cycles) was used to run 75-bp paired-end sequencing.

### Immunofluorescent quantification

Zen Pro 2012 (Carl Zeiss) and ImageJ 1.49v (NIH) software packages were used to process the images. For immunofluorescence quantification, the fluorescence intensity of the microscopic pictures was analyzed using ImageJ for immunofluorescence quantification. Four microscopic pictures were taken per section. The average background intensity of 3 measurements was subtracted from each image. A total of 15 cells per section were selected using the freehand drawing tool, and the area and integrated density were measured. The following formula was used to obtain the fluorescence intensity: fluorescence per pixel = total integrated density/total area.

### Data processing and statistical analysis

The raw sequencing data was aligned to the mm10 genome using the STAR v2.4.0 h aligner with the default settings. Duplicate reads were marked using the MarkDuplicates tool in picard-tools-1.8.4 and removed. The uniquely aligned reads were then counted against Gencode’s GRCm38.p3 GTF annotations using htseq-count in the intersection-strict mode. Final read count files were generated with HTSeq-count version 0.6.1p1. Data analysis of mapped counts was performed in R 3.2.3 using the DESeq2 package (version 1.10) [27]. Samples with less than 6000 genes with at least 5 mapped reads were excluded from analysis (*n* = 0). For unsupervised clustering, sample read counts were normalized using the regularized logarithm transformation method, which is similar to log_2_ transformation for genes with high counts and shrinks together the values for low count genes [27]. The regularized logarithm (rlog) values were used to plot heatmaps using the gplots (version 2.17) heatmap.2 function in R. Unsupervised clustering was performed based on the top 750 most variable genes between samples. Differential expression analysis was performed in DESeq2, and only two-sided Benjamini and Hochberg multiple testing adjusted *p* values are reported in this manuscript. The level of significance used is < 0.05 Benjamini and Hochberg multiple testing adjusted *p* value. Error bars display mean ± standard error of the mean (SEM). The “n” represents three individual mice for the EV-GFP^pos^ microglia and GFP^neg^ tumor and control microglia experiments.

For analysis of specific gene sets, the microglial sensome was extracted from Hickman et al. ^8^. The human sensome was derived in a similar manner as the mouse sensome (manuscript in preparation). The IL6/STAT3 and TGF-β sets were extracted from the gene set enrichment analysis (GSEA) hallmark collection [28]. The IL4, IL10, and IFNγ sets were calculated from the Xue et al. [29] study by extracting the 150 highest upregulated genes compared to baseline. For the IL6/STAT3, TGF-β, IL4, IL10, and IFNγ sets, human to mouse homolog conversions were performed using The Jackson Laboratory Human and Mouse Homology Report (accessed February 18th 2016) supplemented by manual curation. Venn diagrams were generated using the VennDiagram R package (version 1.6.16) [30]. Principal component analysis (PCA) was performed by utilization of the DESeq2’s built-in PCA function using the default settings. Final bar graph, dotplots, PCA, and MA plots were generated in GraphPad Prism (version 5.0c and 7.02).

### Statistical analysis of human glioblastoma macrophage/microglia data

Data on bulk human glioblastoma macrophages/microglia was downloaded from the NCBI Gene Expression Omnibus (GSE80338) as deposited by Szulzewsky et al. [31]. For comparative expression analysis, only samples from glioblastoma patients (*n* = 8) and postmortem controls (*n* = 5) were used. Samples with less than 6000 genes with at least 5 mapped reads were excluded from analysis (*n* = 0). The sample-to-sample heatmap was generated using the Pheatmap R package version 1.08 and using the Eucladian distance between samples.

Single-cell glioblastoma microglia data was extracted from http://www.gbmseq.org/ and described and published by Darmanis et al. [32]. Similar to the original publication, every cell in the myeloid clusters were allocated to the subgroup of either macrophage or microglial origin, based on the mean expression of macrophage (CRIP1, S100A8, S100A9, ANXA1, and CD14) or microglia (TMEM119, P2RY12, GPR34, OLFML3, SCL2A5, SALL1, and ADORA3) markers [32]. For every microglia cell, t-distributed stochastic neighbor embedding (tSNE) mapping was performed based on the published coordinates for every cell in the dataset using ggplot2 version 2.2.1. The normalized read counts and differential expression data were extracted for every microglial cell comparing glioblastoma core cells to peripheral cells using DESeq2 similar as described above.

## Results

### Diffuse microglia, monocytes, and macrophage infiltration in glioblastoma

**Fig. 1:**
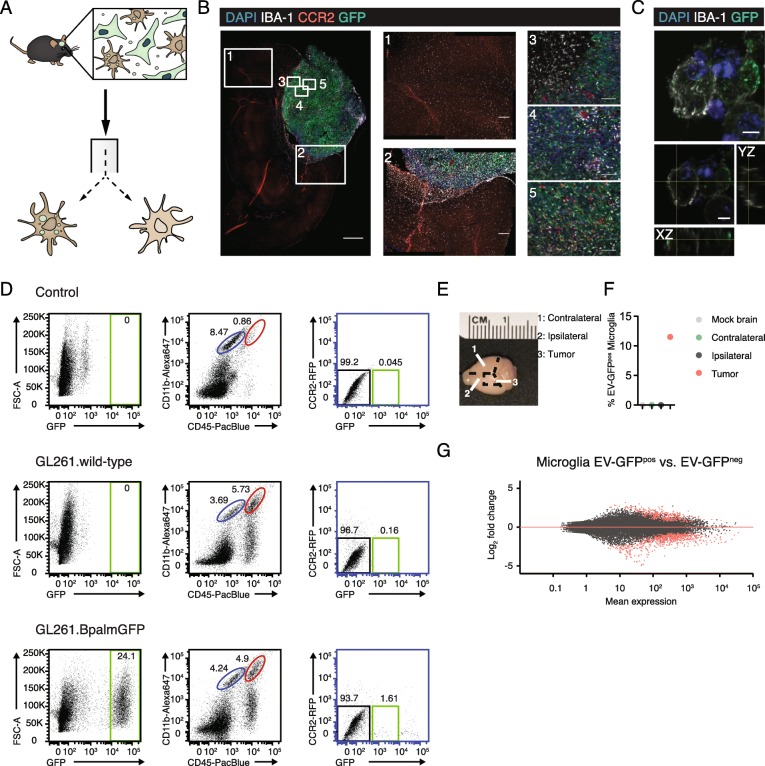
Glioblastoma-interacting microglia internalized tumor-derived GFP. **a** A schematic illustrating our model of C57BL6.CCR2^RFP/WT^ mice implanted with GL261.BpalmGFP glioblastoma cells. Four weeks after tumor implantation, brains were harvested, and microglia, monocytes, and macrophages were sorted based on cell specific antigens and GFP uptake. **b** IBA-1 positive microglia were present throughout the brain (1) and infiltrated the GFP-positive tumor (2–5). CCR2-positive (RFP-labeled) myeloid-derived cells infiltrated the tumor, but were mostly absent in other parts of the brain (1). **c** Confocal microscopy images show that GFP was taken up by IBA-1 positive microglia. **d** Microglia were identified as CD11b^high^/CD45^med^ cells (dark blue gate). Microglia were then sorted based on the GFP signal detected as the upper limit in the control (no tumor) and GL261 wildtype (no GFP) implanted mice. Only in mice implanted with GL261.BpalmGFP, a population of GFP-positive microglia, was identified (green gate in the GFP/CCR2 plot). **e** Delineation of brain areas separated for microglial isolation in F. **f** Only microglia isolated from the tumor contained GFP. Results from a representative experiment are shown. **g** MAplot shows 384 significantly up- or downregulated genes plotted in red when comparing GFP^pos^ (glioblastoma-interacting microglia-GIM) to GFP^neg^ microglia. Scale bars **b** 1000 μm, 1–2; 200 μm, 3–5; 100 μm (**c**), 5 μm

To identify immune cells that had taken up tumor-derived GFP and thus interacted with the tumor (Fig. 1a), we implanted syngeneic mouse glioblastoma cells, GL261.BpalmGFP, or carrier medium in adult C57BL6.CCR2^RFP/WT^ mice that express red fluorescent protein (RFP) under the CCR2 promoter in peripheral blood monocytes and monocyte-derived macrophages, but not in microglia [21]. Four weeks following implantation, the mice were euthanized, and the brains used either for immunofluorescent staining of brain sections or for FACS of brain cells. Using this model, tumor cells express GFP, microglia are labeled with antibodies to IBA-1, and recruited monocytes and macrophages express RFP (Fig. 1b, c). Microglia, monocytes, and macrophages that are closely interacting with glioblastoma cells are positive for IBA-1 and RFP respectively (Fig. 1b). Confocal microscopy and 3-dimensional reconstruction confirmed that GFP is found inside these IBA-1^pos^ microglia (Fig. 1c).

For FACS, we generated highly enriched microglia, monocyte, and macrophage populations from the brains of tumor-bearing and control mice using an established protocol for cell dissociation, isolation, and analysis [26, 33, 34]. Microglia were sorted based on levels of CD11b and CD45 (Fig. 1d). Monocytes and macrophages were separated by additional staining for F4/80 and LY6C, as well as by expression of CCR2-RFP (not shown**)** [21, 35, 36]. The cells were isolated from brains injected with only carrier fluid (control), GL261, or GL261.BpalmGFP tumor cells. Microglia, monocytes, and macrophages were then sorted based on their level of GFP fluorescence to separate cells that had taken up tumor-derived membranous material from those that had not (Fig. 1d). The GFP cutoff was determined by comparing the relative GFP intensity detected in our target cell subsets isolated from brains injected with GL261 wildtype (no GFP) to brains injected with GL261.BpalmGFP (Fig. 1d). By separately analyzing the tumor area, as well as the remaining ipsilateral and contralateral sides of the brain (Fig. 1e**)**, we found that EV-GFP positive (GFP^pos^) microglia, monocytes, and macrophages were only present within and immediately adjacent to the tumor, confirming that the GFP^pos^ cells are closely associated with tumor cells (Fig. 1f). Total RNA was isolated, and sequencing libraries were made using SMARTer Ultra Low Input RNA Kit. Sequencing was done using an Illumina NextSeq, and bioinformatic analysis was performed using DESeq2 in R [27].

### An interactive online tool for analysis of gene expression in microglia, monocytes, and macrophages in glioblastoma

Using this approach, we generated a comprehensive dataset with comparative transcriptomes of control microglia (carrier-injected mice), GFP^pos^ glioblastoma-interacting microglia (EV-GFP^pos^ microglia), and glioblastoma GFP^neg^ microglia. To facilitate analysis of these datasets, we developed an interactive online tool with additional functional and pathway information linked to every gene. The microglia dataset is accessible at http://www.glioma-microglia.com.

To illustrate the usefulness of our dataset, we performed an in-depth analysis of the microglia data and include it in this manuscript. Normalized expression counts and differential expression data available for all genes passing quality metrics are available in Supplementary Table S1a. When analyzing the highest expressed genes in the control, GFP^neg^ microglia, and EV-GFP^pos^ microglia, multiple established microglia genes such as *Cx3cr1*, *HexB,* and *P2ry12* were among the most highly expressed [26, 34] (Supplementary Table S1A). To determine if differential RNAseq expression correlated with differential protein levels, we performed immunofluorescent staining for IBA-1, CD74, and ARG1 comparing the level in microglia from control brains versus tumor-bearing brains and the gene expression level of these genes in the differently sorted microglia populations. Similar levels of IBA-1 protein and RNA levels were detected comparing control versus tumor microglia (Supplementary Fig. 1A-C). In parallel, elevated levels of *Cd74* and *Arg1* RNA in tumor microglia was also detected at the protein expression level of CD74 and ARG1 protein (Supplementary Fig. 1A-C). Overall, these results showed a strong correlation between RNA and protein levels.

### EV-GFP^pos^ microglia represent the most influenced tumor-associated microglia

**Fig. 2:**
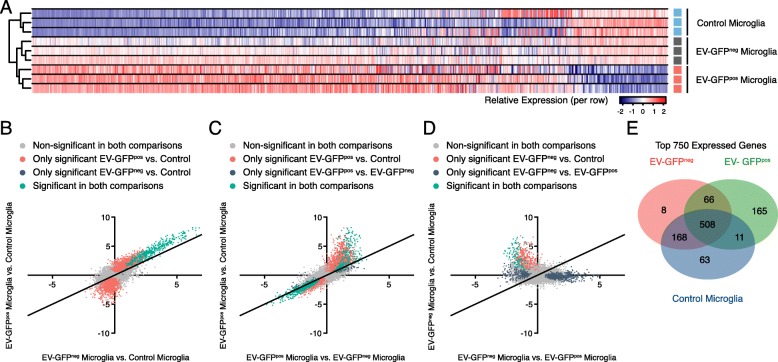
RNA Expression changes are most pronounced in EV-GFP^pos^ microglia compared to GFP^neg^ microglia. **a** In unsupervised clustering of the top 750 most variable genes, microglia cluster together based on tumor status and GFP uptake status. **b** Comparative analysis of differential expression levels of EV-GFP^pos^ microglia and GFP^neg^ microglia compared to control microglia showed 380 shared significantly upregulated genes (green). Overall, the differential expression was higher for EV-GFP^pos^ microglia. These microglia expressed 1426 significantly upregulated and 1196 downregulated genes (red). **c** Most genes significantly changed between EV-GFP^pos^ microglia and GFP^neg^ microglia and were also significantly altered in EV-GFP^pos^ microglia compared to control. **d** These patterns were confirmed in the comparisons of GFP^neg^ to either GFP^pos^ or control. **e** Venn diagram showed overlap between top 750 expressed genes. GFP^neg^ tumor microglia shared most genes with control microglia and EV-GFP^pos^ microglia. This confirmed that EV-GFP^pos^ microglia represent the most altered tumor-associated phenotype

Unsupervised clustering of the top 750 most differentially changed genes showed a clear separation of microglia from control versus tumor-bearing mice, as well as a separation based on GFP status of microglia in tumor-bearing mice (Fig. 2a). When plotting levels of expression for all genes, comparing expression of GFP^neg^ microglia and EV-GFP^pos^ microglia versus control microglia, we found that for most genes, differential expression was stronger for EV-GFP^pos^ microglia than GFP^neg^ versus control microglia (Fig. 2b). Expression of 380 genes was significantly changed in both GFP^neg^ microglia and EV-GFP^pos^ microglia compared to control microglia. In contrast, 2242 genes were significantly changed only in EV-GFP^pos^ microglia (but not in GFP^neg^ microglia) compared to control (Fig. 2b). Comparison of differential expression between EV-GFP^pos^ microglia versus GFP^neg^ or control microglia showed that most genes that are significantly altered in GFP^pos^ versus GFP^neg^ microglia are also significantly changed in EV-GFP^pos^ microglia versus control microglia (Fig. 2c). Comparing GFP^neg^ microglia to either EV-GFP^pos^ microglia or control microglia confirmed these results (Fig. 2d). Evaluation of overlap between the top 750 genes expressed by the three sets of microglia showed most uniquely expressed genes in either control or EV-GFP^pos^ microglia, with GFP^neg^ microglia being in-between (Fig. 2e). This analysis indicates that EV-GFP^pos^ microglia represent a subset of microglial cells that are the most influenced by the tumor.

### Cytokine pathways

The concept that microglia are activated to either an “M1” (INFγ stimulated) or “M2” (IL4 stimulated) state is actively debated in the current literature [37, 38]. We analyzed our dataset to determine if glioblastoma affects microglial cytokine pathways in vivo. We focused on the four pathways regulated by IL4, IL10, IL6/STAT3, and INFγ (Supplementary Figure 2). Overall, analysis of the cytokine signatures in our dataset shows that several tumor-supportive genes belonging to multiple cytokine-related pathways are upregulated in glioblastoma-associated microglia in vivo indicating a more complicated profile than the binary M1/M2 classification. Correlation between gene expression, protein levels, and microglial functions should therefore be performed to determine the in vivo Microglia_Glioblastoma_ specific phenotype.

### Effect of glioblastoma cells on genes involved in key microglial functions

Microglia are involved in brain development, aging, response to injury, and various pathological conditions [38–40]. Microglia have three major functions. First, they continuously survey their milieu to sense changes in their environment. Second, they help protect the brain from invading pathogens and noxious stimuli [41]. Third, they promote homeostasis and synaptic remodeling in development and learning [38, 42]. Microglia express clusters of genes that allow them to perform their different functions and have a number of distinct transcriptomic signatures, which vary with the physiological and/or pathological state of the brain [26, 34]. The homeostatic functions of microglia and expression of genes involved in these functions are regulated by TGF-β [43, 44]. To determine the effects of glioblastoma cells on the three essential microglial functions, we mined our dataset for genes and pathways involved in each of these functions.

### Homeostasis

**Fig. 3:**
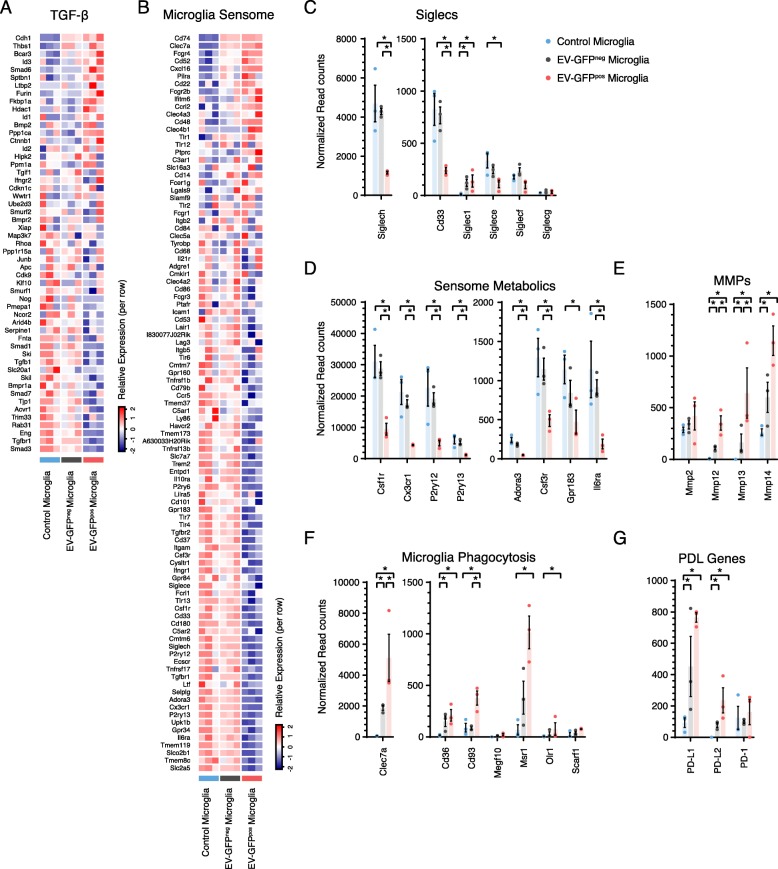
Glioblastoma microglia have a downregulated homeostatic TGF-β pathway, tumor-derived danger signal sensing capacity, and disrupted host defense. **a** TGF-β is the key regulator for microglial homeostasis. In GIM, *Tgfb1* and downstream signaling genes including *Smad3* are significantly downregulated, indicating a disruption of homeostatic functions. **b** EV-GFP^pos^ microglia showed significantly reduced levels of 57% of microglial sensome genes compared to GFP^neg^, indicating reduced capability of sensing of tumor cells and tumor-derived danger signals in EV-GFP^pos^ microglia. **c** Normalized read counts of Siglecs, involved in direct glioblastoma-microglial cellular interactions, showed significant downregulation of *Cd33*, *Siglece*, and *Siglech* in (GFP^pos^) GIM, whereas only *Siglec1* was upregulated. **d** Seven out of eight sensome genes involved in the sensing of metabolic signals were significantly downregulated in EV-GFP^pos^ microglia. **e** Matrix metalloproteinases (MMPs) were upregulated in GIM. *Mmp12*, *Mmp13*, and *Mmp14* were significantly upregulated tumor supportive genes. **f** Genes involved in phagocytic activity in microglial cells were upregulated. *Cd93* and *Clec7a* were significantly higher in EV-GFP^pos^ microglia than GFP^neg^ microglia. **g** Programmed death ligand 1 and 2 (*Pd-l*1 and *Pd-l*2) were significantly upregulated in tumor-associated microglia. Asterisk (*) indicates significant (multiple testing adjusted *p* value < 0.05) differential expression. Error bar represents the SEM, bar represents the mean, and dots display individual measurements (C-G *n* = 3)

TGF-β regulates the microglial homeostatic phenotype [44, 45]. We found that only in GFP^pos^ microglia both *Tgf-β1* and the Tfg-β receptor 1 (*Tgf-βr1*) are significantly downregulated compared to control microglia (log_2_ fold-change − 1.00 and − 2.11, respectively) (Supplementary Table S1B). A global view of the TGF-β pathway revealed that 64.2% of TGF-β genes are downregulated when comparing EV-GFP^pos^ microglia to control microglia (Fig. 3a and Supplementary Table S1B**)**. *Smad3*, one of the key downstream effectors in the TGF-β pathway, is also significantly downregulated in EV-GFP^pos^ microglia (log_2_ fold-change − 2.10) (Fig. 3a and Supplementary Table S1A). Overall, these data imply that TGF-β signaling is downregulated in EV-GFP^pos^ microglia suggesting a disruption in microglial homeostasis.

### Host defense

The third important microglial function is host defense against viral, bacterial, fungal, and parasitic infections, but also against tumor cells [46]. We mined our dataset for microglial genes involved in this function. Interactions of the programmed cell death 1 receptor (PD1) on activated T cells with its ligands programmed death ligand 1 and 2 (PD-L1 and 2) maintain immunologic tolerance through the suppression of auto-reactive T cells [47]. PD-L1 and PD-L2 are expressed on antigen-presenting cells, as well as on tumor cells including glioblastoma [48, 49]. As expected, very little *Pd1* RNA was expressed in microglia as it is usually expressed on T cells [50]. However, increased expression of *Pd-l1* and *Pd-l2* transcripts was higher in EV-GFP^pos^ microglia as compared to GFP^neg^ microglia with both being significantly higher than for control microglia (Fig. 3g). These data identify another pathway by which glioblastoma can possibly evade the immune system, by altering microglia to suppress T cell activation through modulation of T cell immune checkpoints. This finding gains added importance as PD1/PD-L1 directed immune checkpoint therapy is being used against a number of peripheral tumors [51].

### Sensing

The ability to sense changes in the cellular environment in the brain is a major microglial function that allows these cells to adapt to and influence the changing milieu [52, 53]. The armamentarium of 100 genes that allow microglia to perform such functions is termed the sensome [26] (Supplementary Table S1A). These include pattern recognition receptors (25%), receptors involved in cell-cell interaction (10%), chemoattractant and chemokine receptors (10%), cytokine receptors (10%), Fc receptors (7%), purinergic receptors (8%), receptors for extracellular matrix (ECM) proteins (6%), other receptors or transporters (13%), and potential sensome proteins with no known ligands (11%) [26]. When analyzing expression levels of genes involved in microglial sensing, we identified overall downregulation of the sensing capacity in glioblastoma-interacting microglia (Fig. 3b).

Sensome transcripts that were downregulated in EV-GFP^pos^ microglia compared to GFP^neg^ and control microglia can be divided into three groups. Group one includes transcripts encoding proteins that directly mediate microglia-glioblastoma cellular interactions. Indeed, sialic acid-binding immunoglobulin-like lectin-H (*Siglech*) is a CD33-related Siglec that is a microglial sensor of glioblastoma cells [54]. *Siglech* is significantly downregulated in EV-GFP^pos^ microglia compared to GFP^neg^ and control microglia (log_2_ fold-change − 1.84 and − 1.97, respectively) (Fig. 3c). Interestingly, *Cd33* is also significantly downregulated in GFP^pos^ compared to GFP^neg^ and control microglia (log_2_ fold-change − 1.62 and − 1.72, respectively) (Fig. 3c). It is not known if CD33, like Siglech, is also a sensor of glioblastoma cells. Another microglial receptor that is capable of sensing lysophosphatidylserine exposed on glioblastoma cells is GPR34 [55, 56]. Similar to Siglech, *Gpr34* a gene known to directly sense ligands expressed in glioblastoma cells is downregulated in EV-GFP^pos^ microglia compared to GFP^neg^ and control microglia (log_2_ fold-change − 1.96 and − 2.37, respectively) (Supplementary Table S1A). These data indicate that EV-GFP^pos^ microglia, but not other microglia in the same tumor-bearing brain, have reduced expression of at least two transcripts, encoding the proteins SIGLECH and GPR34, known to directly sense ligands expressed on glioblastoma cells.

A second group of transcripts that is downregulated in EV-GFP^pos^ microglia, but not in GFP^neg^ microglia, includes those encoding proteins that sense metabolic products potentially released by glioblastoma cells. These transcripts include *Gpr183*, *Adora3*, *Il6Ra*, *Cx3cr1*, *P2ry12*, *P2ry13*, *Csf1r*, and *Csf3r* (Fig. 3d). GPR183 is a sensor for oxysterols, which are released by glioblastoma cells and play a role in recruitment of immune cells [57]. ADORA3 is a sensor for adenosine that is released by glioblastoma cell ectonucleotidases. Adenosine promotes tumor growth, can activate toll-like receptors (TLRs), and induces microglial responses via an ADORA3-dependent mechanism [58]. IL6Ra is a receptor for IL6, with elevated levels of IL6 in glioblastomas associated with poor survival in patients [59]. Expression of the fractalkine receptor CX3CR1 was also decreased, and loss of CX3CR1 has been shown to promote glioblastomagenesis [60]. P2RY12 and P2RY13-purinergic receptors for ATP, which is an important signaling molecule in the CNS, are both down (Fig. 3d). This could promote tumor growth by two different pathways. First, necrosis, one of the hallmarks of glioblastoma, liberates nucleotides into the extracellular milieu. These nucleotides are hydrolyzed very slowly by glioblastomas and induce neuronal cell death and glioblastoma proliferation [61]. Second, extracellular ATP activates microglial P2RY12 receptors that are utilized to trigger an acute inflammatory response in microglia via rapid CCL3 induction after ADP stimulation [62]. Therefore, downregulating microglial receptors for ATP could preserve the ability of the nucleotides to promote tumor growth, while reducing the ability of microglia to respond to the tumor, thereby further enhancing the tumor’s advantage.

Of note, the overall expression pattern of all 100 sensome pathway genes showed differential gene expression in only 4% of sensome genes when comparing GFP^neg^ to control microglia (Fig. 3b and Supplementary Table S1B). These genes (e.g., *Cd74*, *Clec7a*, *Cxcl16*, and *Fcgr4*) were all upregulated compared to control microglia. In contrast, we found significant changes in gene expression between EV-GFP^pos^ microglia versus GFP^neg^ and control microglia in 57% of sensome transcripts. Remarkably, 48% of sensome genes were downregulated in GFP^pos^ microglia and only 9% upregulated (Fig. 3b). This could indicate that the microglia infiltrated into glioblastoma are not able to sense the tumor. Overall, while further research is required to validate the exact impact of individual sensome genes on tumor growth, our results show that microglia dramatically change their expression profile in the presence of a tumor, reducing their capacity to sense changes in the (tumor) microenvironment.

### Pathways involved in tumor growth

Since the expression of genes involved in the maintenance of homeostasis within EV-GFP^pos^ microglia are disrupted, we investigated the effects of this disruption on three pathways that maintain brain homeostasis and affect tumor growth. The role of microglia in maintaining brain homeostasis includes debris breakdown and removal by matrix metalloproteases (MMPs) [26]. MMP enzymes could also play an important role in promoting tumor growth by making space for tumor cells to migrate, invade, and proliferate [3, 9, 10]. In glioblastoma, MMP2 serves as an important MMP to degrade the extracellular matrix (ECM) subsequently enabling the invasive properties of glioblastoma [63]. MMP2 is secreted by glioblastoma cells in a pro-form (pro-MMP2) which needs to be cleaved by *Mmp14* (MT1-MMP) to be active [9, 10]. Tumor microglial cells are an important source of MMP14 [9, 10]. Previously, we showed that *Mmp14* levels are increased in glioblastoma-associated microglia in vitro [17]. *Mmp14* was among the three Mmps (*Mmp12*, *Mmp13*, and *Mmp14*) that were significantly upregulated in EV-GFP^pos^ microglia and to a lesser extent in GFP^neg^ microglia (Fig. 3e**)**. These data indicate that glioblastoma alters microglial gene expression patterns in a manner that could favor tumor spread and migration by clearing debris and digesting the ECM in the tumor microenvironment.

In addition to changes in *Mmps*, we also found that glioblastoma was associated with an increased expression of mRNAs encoding microglial phagocytic receptors—*Cd93*, *Msr1*, *Cd36*, *Olr1*, *Megf10*, *Clec7a*, and *Scarf1* (Fig. 3f**)**. The roles of these phagocytic receptors in promoting debris clearance and subsequent tumor growth have not yet been investigated. However, since these receptors promote clearance of apoptotic cells [64], it is plausible that these receptors, in conjunction with MMPs, promote the phagocytic clearance of debris in the tumor environment further facilitating tumor spread.

### Microglial uptake of EVs is associated with decreased sensome expression

**Fig. 4:**
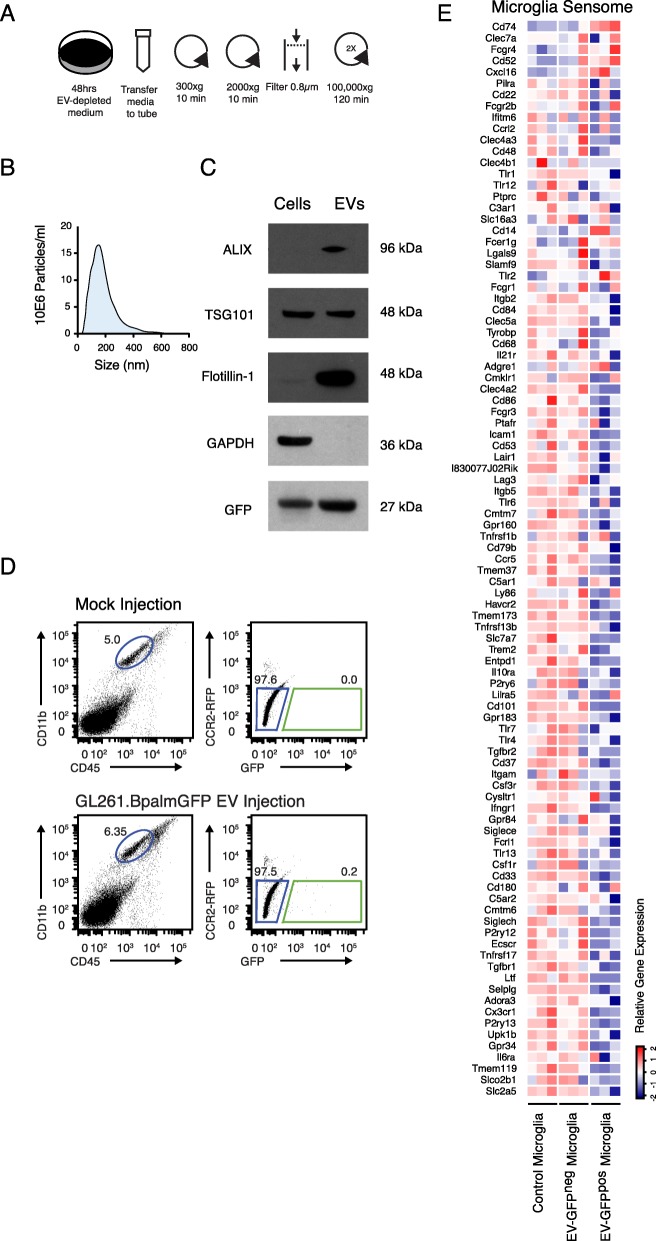
Uptake of intracranially injected glioma-derived fluorescent EVs is associated with a decrease in microglial sensing capability. **a** Schematic overview of EV isolation from glioma cells in culture using differential centrifugation. **b** Size distribution analysis using NTA of isolated EVs shows small and larger vesicles present in the EV preparation. **c** Western blot analysis shows GFP present in cells and EV, and extracellular vesicles markers (ALIX, TSG101 and Flotillin-1) enriched in vesicles lysate and GAPDH is detected in cellular lysate only. **d** Microglia were identified as CD11b^high^/CD45^med^ cells (blue gate). Microglia were then sorted based on the GFP signal detected as the upper limit in control. In mice injected with GL261.BpalmGFP EVs, a population of GFP-positive microglia was identified (green gate in the GFP/RFP plot). **e** Heatmap of sensome genes ordered top to bottom by highest up- to downregulated for mouse EV-GFP^pos^ tumor microglia compared to wildtype (same order as Fig. 3b). Similar patterns are observed for genes up- and downregulated compared to the mouse tumor-derived profile. Asterisk (*) indicates significant (multiple testing adjusted *p* value < 0.05) differential expression. Error bar represents the SEM, bar represents the mean, and dots display individual measurements (*n* = 3)

To explore the relationship between microglial uptake of glioblastoma derived EVs and the expression of sensome genes, we evaluated RNA expression by microglia isolated from control (non-tumor bearing) C57BL6.CCR2^RFP/WT^ mice injected with carrier fluid or with EVs isolated from GL261.BpalmGFP cells. EVs were isolated using standard step-wise (ultra)centrifugation (Fig. 4a) and as expected, the isolated EVs were within the 80–400-nm size range (Fig. 4b) expressing the EV associated proteins ALIX, TSG101, and Flotillin-1 as well as GFP (Fig. 4c). Sixteen hours after EV injection, microglia were isolated based on EV uptake and their transcriptomes analyzed by RNASeq (Fig. 4d). Similar to the results from EV-GFP^pos^ microglia isolated from tumor bearing brains, overall downregulation of the microglia sensome genes was observed in microglia that took GFP-EVs injected into the brain (Suppl. Table S2 and Fig. 4e). It is possible that some of the changes observed in EV-GFP^pos^ microglia did not reach significance because the number of EVs added and time point of analyses may bias the result. These data show parallels between tumor microglia and microglia isolated after EV-injection and open the door for further investigation of specific EV contents that may induce the changes observed.

### Human glioblastoma-associated microglia have a reduced sensing capacity

**Fig. 5:**
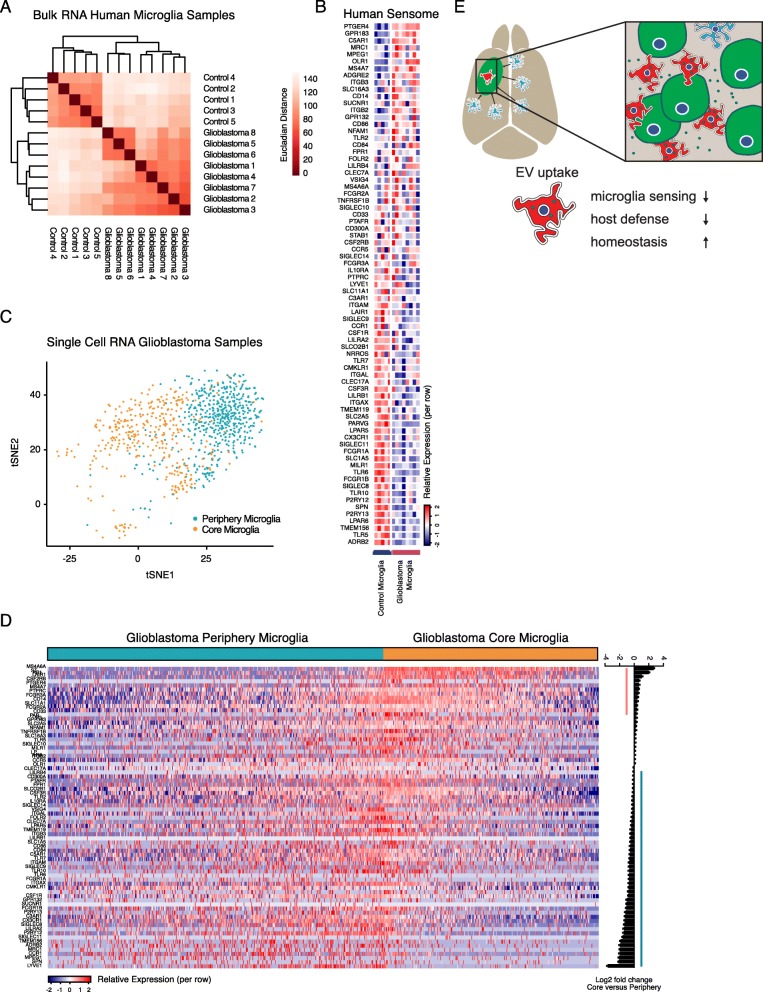
The Sensome is downregulated in human microglia from glioblastoma patients. **a** Analysis of published bulk RNAseq data from CD11B^pos^ microglia harvested from postmortem human brains (control) or glioblastoma patients identifies differences based on sample group as well as heterogeneity between glioblastoma derived cells. **b** Glioblastoma microglia showed significantly reduced levels in 32% of genes versus 12% upregulation, indicating reduced overall capability of sensing of tumor cells and tumor-derived danger signals in human glioblastoma microglia. Further analysis of published human glioblastoma single-cell microglia data identified similar results. **c** Expression levels of TMEM119, P2RY12, GPR34, OLFML3, SCL2A5, SALL1, and ADORA3 for microglia and CRIP1, S100A8, S100A9, ANXA1, and CD14 for macrophages were used to identify individual microglia and macrophages cells isolated at either the core or periphery of the glioblastoma mass. **d** At a single-cell level, 15% of genes are significantly upregulated (genes in red), and 48% of the human sensome genes are significantly downregulated (genes in blue) when comparing microglia at the core to microglia in the periphery of the glioblastoma mass again indicating reduced capability of sensing of tumor cells and tumor-derived danger signals in human glioblastoma microglia. **e** Schematic illustration showing the anti-tumor ability of microglia after EV uptake by simultaneous reduction of the sensing capacity and host defense as well as an increased homeostatic function. This pathway is ultimately required for glioblastoma growth. Asterisk (*) indicates significant (multiple testing adjusted *p* value < 0.05) differential expression. Error bar represents the SEM, bar represents the mean, and dots display individual measurements (**a**, **b**: control n = 5, glioblastoma *n* = 8, **C**, **D** microglia core *n* = 365, microglia periphery *n* = 574)

To determine if changes in gene expression in human glioblastoma-associated microglia are similar to those observed in mouse microglia, we analyzed two existing published datasets of human microglia. These datasets contain bulk RNA sequencing results comparing post-mortem brains (controls) to CD11b^pos^ macrophage/microglia isolated from glioblastoma samples (GEO Accession GSE80338) [31] and single-cell RNA sequencing data comparing microglia isolated from either the core or the periphery of the glioblastoma tumor mass (data from http://www.gbmseq.org/) described and published by Darmanis et al. [32]. As expected, the control and glioblastoma-associated cells cluster separately with some heterogeneity within the glioblastoma samples (Fig. 5a). Similar to our mouse samples, 32% of human microglial sensome genes were downregulated, and only 12% were upregulated in human glioblastoma microglial cells compared to control, paralleling our data obtained from mice (Fig. 5b).

We then assessed if these results could be confirmed using published single-cell microglia data from human patients with glioblastoma. These data were obtained from microglia isolated either from the core of a glioblastoma tumor or the periphery [32]. Since microglia within the tumor mass are more likely to interact directly with tumor cells than microglia from the periphery of the tumor, we hypothesized that microglia from the human tumor core will most likely resemble mouse GIM and will have similar glioblastoma-induced RNA expression to mouse GFP^pos^ microglia. To separate microglia from macrophages in the dataset, we used the expression levels of TMEM119, P2RY12, GPR34, OLFML3, SCL2A5, SALL1, and ADORA3 as microglial markers and CRIP1, S100A8, S100A9, ANXA1, and CD14 as macrophage markers [26]. By focusing on the identified microglia only, we could see clear separation of microglial cells isolated from the core or periphery of the tumor (Fig. 5c). Similar to our results from mice, the microglia isolated from the core of human glioblastoma have a reduced sensing capacity with significantly reduced expression of 48% of sensome genes versus only 15% upregulation (Fig. 5d).

Taken together, these data identify reduced expression of microglia sensing genes in glioblastoma microglia suggesting reduced sensing capacity in these cells (Fig. 5e).

## Discussion

Glioblastomas are the most aggressive malignant brain tumors leading invariably to death. To date, no effective therapy has been found for this devastating disease. These tumors are heavily infiltrated with innate immune cells including resident brain microglia. Yet, despite such a large immune cell presence, glioblastomas continue to grow and are thought to co-opt the innate immune system of the host to promote tumor spread [3]. To determine how glioblastoma affects the innate immune system, we analyzed the gene expression profile of microglia in a mouse model of this tumor using RNA sequencing. By using glioblastoma cells with fluorescently labeled membranes, we could identify and separate microglial cells closely associated with the tumor by their uptake of tumor-derived fluorescent membranes/membrane particles including EVs (EV-GFP^pos^) from those EV-GFP^neg^ microglia that were further away from the tumor. We compared EV-GFP^pos^ and EV-GFP^neg^ microglia with each other and with microglia isolated from normal brains. Our data show that EV-GFP^pos^ glioblastoma microglia have a unique gene expression profile that distinguishes them from other microglia and that this glioblastoma-associated expression profile is more complex than the prior classification of M1 versus M2 states. Instead, we identified a disease-specific Microglia_Glioblastoma_ state that is characterized by markers found in both M1 and M2 polarization states. This glioblastoma-associated expression profile defines a disease-specific Microglia_Glioblastoma_ state that could be further subclassified based on proximity of the microglia to the tumor. In these Microglia_Glioblastoma_, genes that promote tumor killing are downregulated, whereas genes that promote tumor growth, invasion and immune suppression are upregulated.

We identified at least three pathways by which EV-GFP^pos^ microglia became less effective in combating the tumor and more geared towards promoting tumor growth. First, and most dramatically, we found that EV-GFP^pos^ microglia had reduced expression of genes involved in sensing tumor cells and tumor-derived cellular byproducts. A decreased ability to sense and recognize tumor cells makes these cells “hidden” from the immune system and therefore protected from anti-tumor immune activities. A second group of microglial transcripts altered by interaction with glioblastoma cells reflects a disarming of their usual anti-tumor functions. These include upregulation of PD-L1 and PD-L2 which help maintain immunologic tolerance by causing T cell exhaustion and ultimately reducing the tumor killing capacity of T cells [65]. We also found that microglial genes that suppress cytotoxic T cell activation and those in direct tumor killing, such as antimicrobial peptides are also suppressed [66, 67].

In contrast to reducing microglial tumor sensing and anti-tumor abilities, glioma cells enhance the capacity of microglia to promote tumor spread, by affecting genes that alter the extracellular milieu surrounding tumor cells. One of the hallmarks of glioblastoma is the presence of excessive debris and necrotic tissue, and clearing such necrotic material is important for tumor cell invasion and growth [68]. We found that microglia in the micro environs of tumors have increased expression of several phagocytic receptors, while either maintaining or increasing expression of extracellular matrix degrading enzymes. Clearing debris and necrotic tissue from the tumor milieu would boost the migratory capacity of tumor cells, one of the key characteristics of glioblastoma. These data indicate that glioblastoma-interacting microglia may help promote tumor growth and migration by clearing debris in the tumor microenvironment.

Our novel method of identifying microglia that have taken up tumor-derived EVs in vivo allows us to select microglia with which the tumor appears to have interacted directly with a physical exchange of membrane and cytoplasmic factors. Simultaneously, this could suggest that some of the gene expression changes observed are related to the uptake of EVs. In fact, when comparing microglia that took up glioma EVs in a non-tumor bearing brain to control microglia, we could detect similar gene expression changes as observed in EV-GFP^pos^ tumor microglia. However, in the in vivo tumor model described here, all tumor lipid bilayers are GFP-positive and thus it is not clear whether all GFP^pos^ microglia have taken up EVs per se or may possibly have taken up tumor cell membrane debris. Other intercellular communication modes such as secreted molecules [69], exchange of molecules through gap junctions between cells [70], and cell connecting nano/microtubes may contribute to the observed effects as well. Glioma secreted cytokines (e.g., CSF-1, MCP-3, CX3CL1, SDF-1, and GM-CSF) that are especially known to be involved in the recruitment of microglial cells and could be responsible (in part) for the observed changes in gene expression, with EV-GFP uptake being a mere side-effect [3]

Glioblastomas are heterogeneous tumors at the inter- and intratumor level and they express gene patterns associated with mesenchymal, proneural, and classical subtypes [71]. We recognize that a single, highly clonal, murine glioma line may not recapitulate this heterogeneity. To address this issue, we analyzed existing datasets obtained from human patients with glioblastoma and found that these data support the conclusions obtained with our mouse model and reflect the true heterogeneity of human glioblastoma tumors, further asserting the validity of our analysis and its applicability to human disease.

For the sake of exploratory analysis and to increase the impact of our dataset, we established an online tool accessible at http://www.glioma-microglia.com that includes the microglia dataset. This webtool will facilitate the identification of additional genes associated with these tumors and are a useful tool for discovery.

## Conclusions

Overall, our data open the door for future investigations to specifically identify how glioblastoma hijack the microglial immune response to promote tumor growth and will possibly help identify novel microglia-specific targets for therapy of this highly aggressive and so far, untreatable lethal disease. Our findings indicate that glioblastoma-associated microglia suppress the adaptive immune response to the tumor, have a reduced capacity to directly kill tumor cells, and promote tumor cell invasion and proliferation.

## Supplementary information

**Additional file 1: Table S1.** a, b all genes and subsets.

**Additional file 2: Figure S1.** RNA levels correlated with protein levels in control and tumor-bearing brains. (A) The microglial marker *Iba1* was equally expressed in control and tumor-associated microglia, whereas *Cd74* and *Arg1* expression was increased in tumor-associated microglia as measured by RNAseq. (B) Immunofluorescence staining of IBA1, CD74 and ARG1 in control and tumor-bearing mouse brains. (C) Quantification of immunofluorescent staining seen in (B) Fluorescent intensity was quantified per pixel within all identified cells. Tumor and control tissues were individually compared for each marker. IBA1, CD74 and ARG1 fluorescence quantification correlated with RNA data whereas Scale bars 100 μm, asterisk indicates multiple testing adjusted p-value <0.05, error bar represents SEM.

**Additional file 3: Figure S2.** IL4, IL10, IL6 and IFNγ pathways genes were upregulated in tumor-associated microglia. (A) The IL4 associated genes were mostly upregulated in tumor-associated microglia with increased expression in EV-GFP^pos^ microglia. The significantly upregulated genes in EV-GFP^pos^ versus EV-GFP^neg^ microglia included known tumor supportive genes such as *Mmp12*, *Adam19* and *Wnt5a*. (B) IL10 related genes were upregulated in tumor microglia. *Sod2*, a tumor supportive gene, was among the genes significantly upregulated in EV-GFP^pos^ microglia. (C) IL6 related genes were upregulated in tumor-associated microglia. Among the significantly upregulated IL6 genes is *Ccl7 (MCP-3)*, a secreted chemokine involved in the attraction of microglia and macrophages to the tumor suggesting a tumor supportive infiltration loop. (D) Overall, increased expression of IFNγ related genes was observed with the strongest expression in EV-GFP^pos^ microglia. Among the significantly upregulated genes in EV-GFP^pos^ microglia was *Irf7,* a key regulator of pro-inflammatory to anti-inflammatory switching in microglia.

**Additional file 4: Table S2.** EV injection data all genes and subsets.

## Data Availability

Data availability Raw and processed transcriptomic data described in this manuscript are deposited in NCBI’s Gene Expression Omnibus (GEO) and are accessible using GEO Series accession number GSE106775 at https://www.ncbi.nlm.nih.gov/geo/query/acc.cgi?acc = GSE106775. Token for early data access: wdidoocgxxqjxsn. Code availability R scripts written for data processing and the generation of figures included in this manuscript are available online in a git repository. This includes the R sessionInfo() data for compatibility information. The files and information can be accessed at https://github.com/slnmaas/Glioblastoma-Microglia-Project

## Abbreviations

EVs: Extracellular vesicles
FACS: Fluorescence activated cell sorting
TGF-β: Transforming growth factor beta
DMEM: Dulbecco’s modified Eagle’s medium
FBS: Fetal bovine serum
TSG101: Tumor susceptibility gene 101
GAPDH: Glyceraldehyde 3-phosphate dehydrogenase
GFP: Green fluorescent protein
NGS: Normal goat serum
BSA: Bovine serum albumin
ARG1: Arginase 1
CD74: Cluster of differentiation 74
IBA1: Ionized calcium binding adaptor molecule 1
CD45: Protein tyrosine phosphatase receptor type C (PTPRC)
CD11b: Integrin alpha M (ITGAM)
F4/80: EGF-like module-containing mucin-like hormone receptor-like 1 (EMR1)
Ly6C: Lymphocyte antigen 6C
CCR2: C-C chemokine receptor type 2
PCA: Principle component analysis
MA: M (log ratio) and A (mean average)
RFP: Red fluorescent protein
Cx3cr1: CX3C chemokine receptor 1
HexB: Hexosaminidase subunit beta
Tgf-βr1: Tfg-β receptor 1
PD1: Programmed cell death 1 receptor
PD-L1: Programmed death ligand 1
PD-L2: Programmed death ligand 2
Siglech: Sialic acid-binding immunoglobulin-like lectin-H
Cd33: Sialic acid binding Ig-like lectin 3 (Siglec-3)
Gpr34: G-protein coupled receptor 34
Gpr183: G-protein coupled receptor 183
P2ry12: P2Y purinoceptor 12
P2ry13: P2Y purinoceptor 13
Csf1r: Colony stimulating factor 1 receptor
Csf3r: Colony stimulating factor 3 receptor
ADORA3: Adenosine A_3_ receptor
TLR: Toll-like receptor
IL6Ra: Interleukin 6 receptor alpha
IL6: Interleukin 6
ATP: Adenosine triphosphate
CNS: Central nervous system
CCL3: Chemokine ligand 3
ADP: Adenosine diphosphate
Cxcl16: C-X-C Motif chemokine ligand 16
Fcgr4: Fc gamma receptor 4
MMP: Matrix metallopeptidase
Cd93: Cluster of differentiation 93
Msr1: Macrophage scavenger receptor 1
Cd36: Platelet glycoprotein 4
Olr1: Oxidized low-density lipoprotein receptor 1
Megf10: Multiple EGF like domains 10
Clec7a: C-type lectin domain containing 7A
Scarf1: Scavenger receptor class F member 1
TMEM119: Transmembrane protein 119
OLFML3: Olfactomedin-like 3
SCL2A5: Solute carrier family 2 member 5
SALL1: Spalt-like transcription factor 1
CRIP1: Cysteine-rich intestinal protein 1
S100A8: S100 calcium-binding protein A8
S100A9: S100 calcium-binding protein A9
ANXA1: Annexin A1
CD14: Cluster of differentiation 14
