# Supplementary figures and images for: Glioblastoma hijacks microglial gene expression to support tumor growth
Source: J Neuroinflammation. 2020 Apr 16;17:120. doi: 10.1186/s12974-020-01797-2 (PMC7164149; doi:10.1186/s12974-020-01797-2)

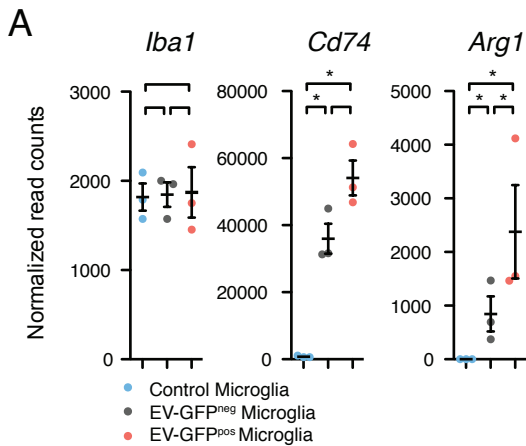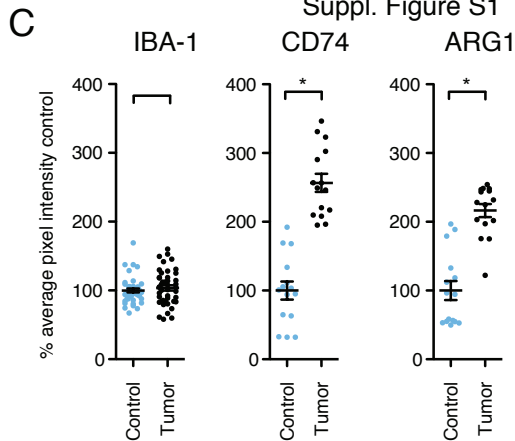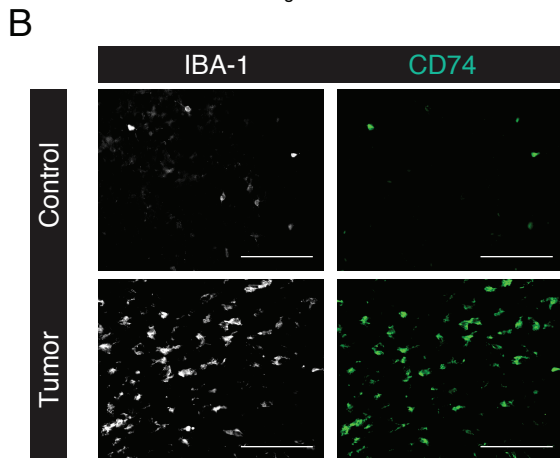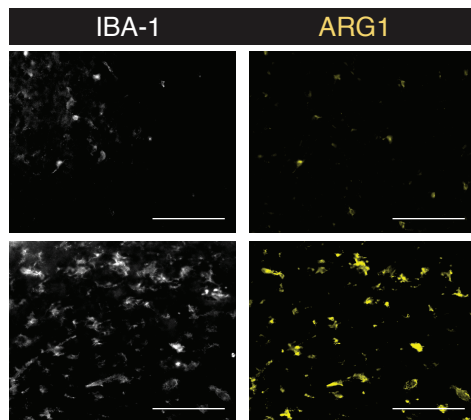

Supplement: Supplementary file 2 — Additional file 2: Figure S1. RNA levels correlated with protein levels in control and tumor-bearing brains. (A) The microglial marker Iba1 was equally expressed in control and tumor-associated microglia, whereas Cd74 and Arg1 expression was increased in tumor-associated microglia as measured by RNAseq. (B) Immunofluorescence staining of IBA1, CD74 and ARG1 in control and tumor-bearing mouse brains. (C) Quantification of immunofluorescent staining seen in (B) Fluorescent intensity was quantified per pixel within all identified cells. Tumor and control tissues were individually compared for each marker. IBA1, CD74 and ARG1 fluorescence quantification correlated with RNA data whereas Scale bars 100 μm, asterisk indicates multiple testing adjusted p-value <0.05, error bar represents SEM. [file 12974_2020_1797_MOESM2_ESM.pdf]

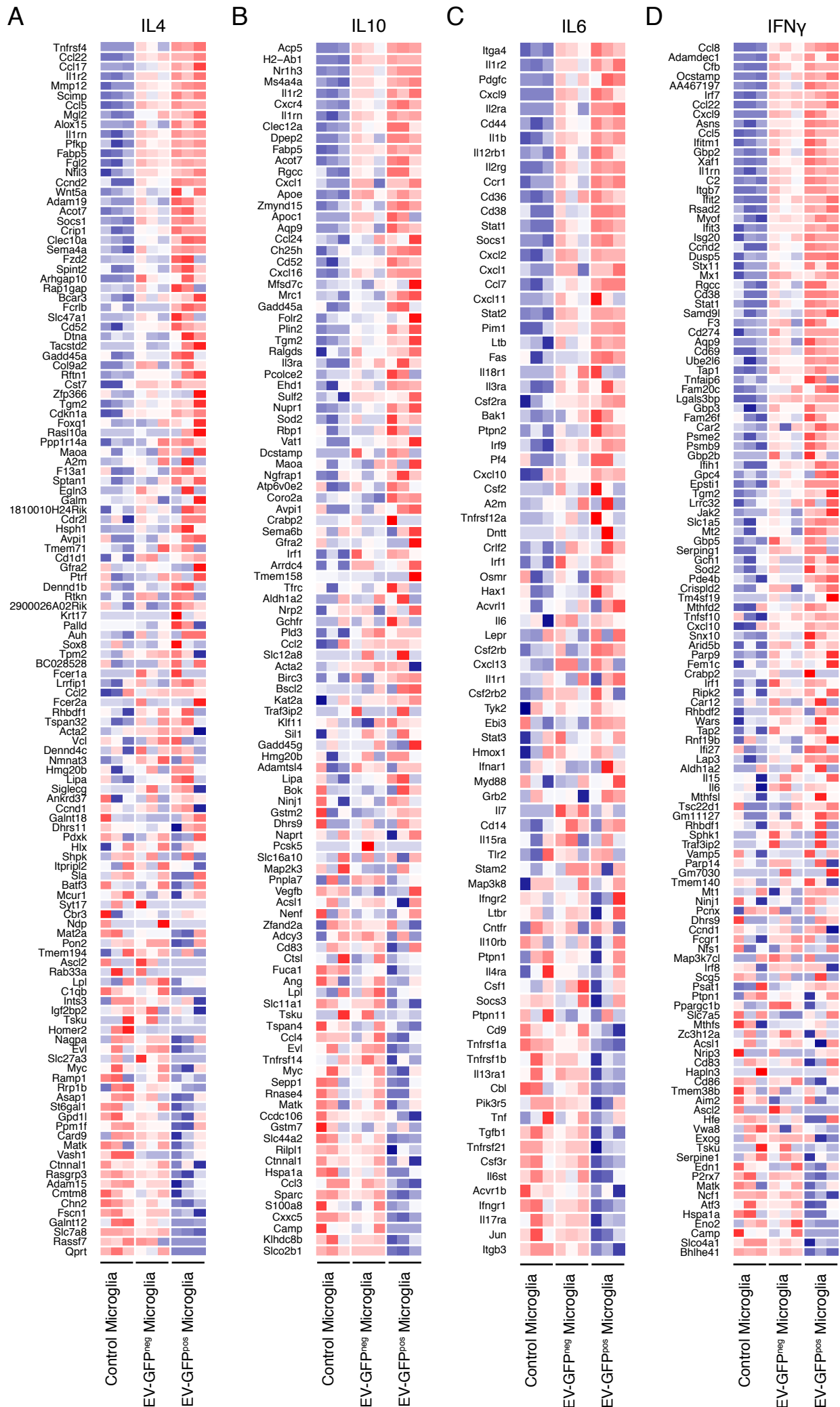

Supp. Fig. S2

Supplement: Supplementary file 3 — Additional file 3: Figure S2. IL4, IL10, IL6 and IFNγ pathways genes were upregulated in tumor-associated microglia. (A) The IL4 associated genes were mostly upregulated in tumor-associated microglia with increased expression in EV-GFPpos microglia. The significantly upregulated genes in EV-GFPpos versus EV-GFPneg microglia included known tumor supportive genes such as Mmp12, Adam19 and Wnt5a. (B) IL10 related genes were upregulated in tumor microglia. Sod2, a tumor supportive gene, was among the genes significantly upregulated in EV-GFPpos microglia. (C) IL6 related genes were upregulated in tumor-associated microglia. Among the significantly upregulated IL6 genes is Ccl7 (MCP-3), a secreted chemokine involved in the attraction of microglia and macrophages to the tumor suggesting a tumor supportive infiltration loop. (D) Overall, increased expression of IFNγ related genes was observed with the strongest expression in EV-GFPpos microglia. Among the significantly upregulated genes in EV-GFPpos microglia was Irf7, a key regulator of pro-inflammatory to anti-inflammatory switching in microglia. [file 12974_2020_1797_MOESM3_ESM.pdf]
